# Engineering of a GSH activatable photosensitizer for enhanced photodynamic therapy through disrupting redox homeostasis†

**DOI:** 10.1039/d3ra04074g

**Published:** 2023-07-25

**Authors:** Datian Fu, Yan Wang, Kaiwen Lin, Liangjiu Huang, Jin Xu, Haimei Wu

**Affiliations:** a Department of Pharmacy, Hainan Women and Children's Medical Center Haikou Hainan 570100 China; b Department of Clinical Pharmacy, Hainan Cancer Hospital Haikou Hainan 570100 China wu634202863@126.com; c Pharmaceutical and Bioengineering School, Hunan Chemical Vocational Technology College Zhuzhou 412006 China

## Abstract

Although disrupted redox homeostasis has emerged as a promising approach for tumor therapy, most existing photosensitizers are not able to simultaneously improve the reactive oxygen species level and reduce the glutathione (GSH) level. Therefore, designing photosensitizers that can achieve these two aspects of this goal is still urgent and challenging. In this work, an organic activatable near-infrared (NIR) photosensitizer, CyI-S-diCF_3_, is developed for GSH depletion-assisted enhanced photodynamic therapy. CyI-S-diCF_3_, composed of an iodinated heptamethine cyanine skeleton linked with a recognition unit of 3,5-bis(trifluoromethyl)benzenethiol, can specifically react with GSH by nucleophilic substitution, resulting in intracellular GSH depletion and redox imbalance. Moreover, the activated photosensitizer can produce abundant singlet oxygen (^1^O_2_) under NIR light irradiation, further heightening the cellular oxidative stress. By this unique nature, CyI-S-diCF_3_ exhibits excellent toxicity to cancer cells, followed by inducing earlier apoptosis. Thus, our study may propose a new strategy to design an activatable photosensitizer for breaking the redox homeostasis in tumor cells.

## Abstract 2

The novel NIR photosensitizer, CyI-S-diCF_3_, with excellent GSH consumption ability and ^1^O_2_ production efficiency, was successfully used for GSH depletion-assisted enhanced photodynamic therapy through disrupting redox homeostasis.

## Graphical abstract

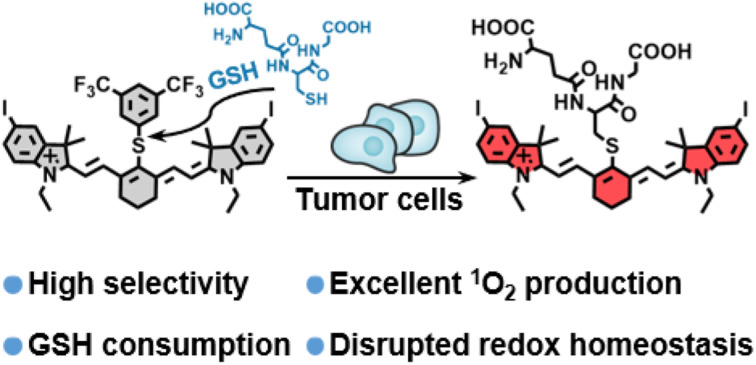

## 1. Introduction

Redox homeostasis, a dynamic balance of reactive oxygen species (ROS) generation and elimination, is a significant index for cell metabolism.^1–5^ ROS (*e.g.*, anion superoxide, hydroxyl radical and singlet oxygen) are usually generated by intracellular oxidoreductase-catalyzed processes and exogenous light stimulation.^6–9^ The elevated ROS could promote the proliferation of tumors, whereas an excessive amount of ROS would elicit cell death due to strengthened oxidative stress.^10,11^ To equilibrate the high-level of oxidative stress, cancer cells would functionally enhance the endogenous antioxidant systems by producing glutathione (GSH), an important reducing agent enriched in tumor tissue.^12–14^ Given this unique feature, breaking the cellular redox homeostasis to induce apoptosis would be recognized as a promising strategy for tumor therapy. Recently, numerous nanomaterials have been constructed to disrupt the redox homeostasis by boosting the ROS level and concurrently decreasing GSH concentrations, leading to cell apoptosis.^15–19^ However, there are still very few organic photosensitizers (PSs) able to simultaneously achieve the amplification of ROS level and down-regulation of GSH content in tumor cells.^20^ Thus, it is of great importance to rationally design and synthesis novel photosensitizers that exhibited excellent ROS generation ability and GSH depletion property, which, however, is still a great challenge.

Owing to its precise spatiotemporal selectivity, noninvasive and biosafety feature, photodynamic therapy (PDT) has regarded as a promising oncotherapy.^21–26^ Photosensitizers are usually able to produce toxic singlet oxygen (^1^O_2_) to kill tumor cells through the energy transfer (type II) process between the triplet state in PSs and surrounding O_2_ during photodynamic therapy.^27–29^ However, though many photosensitizers have been used for PDT, the tumor suppression efficacy was still weak due to the limited photon utilization of the photosensitizers, which could be attributed to the short excitation wavelengths and low molar extinction coefficient.^30^ Furthermore, on account of the aggressive proliferation of cancer cells, intracellular O_2_ concentrations was lower than normal cells, which further restricted the ^1^O_2_ generation efficiency of photosensitizers in phototherapy.^31,32^ To date, several studies have been reported that heptamethine cyanine dyes could be used as an effective photosensitizer for PDT as its near-infrared (NIR) excitation light and high molar extinction coefficient. Meanwhile, the ^1^O_2_ generation efficacy of these PSs could be distinctly improved by accelerating the intersystem crossing (ISC) process using heavy-atom effect (HAE).^33–37^ It is worth noting that the chloride atom at the meso position in heptamethine cyanine skeleton also could be easily modified by different groups to construct functional fluorescence probes (*e.g*., GSH-activatable fluorescence probe).^38–40^ Therefore, heptamethine cyanine scaffold might provide an promising platform to design a novel photosensitizer with enhanced ROS production efficiency and high GSH consumption ability for breaking the redox homeostasis in tumor cells.

**Scheme 1:**
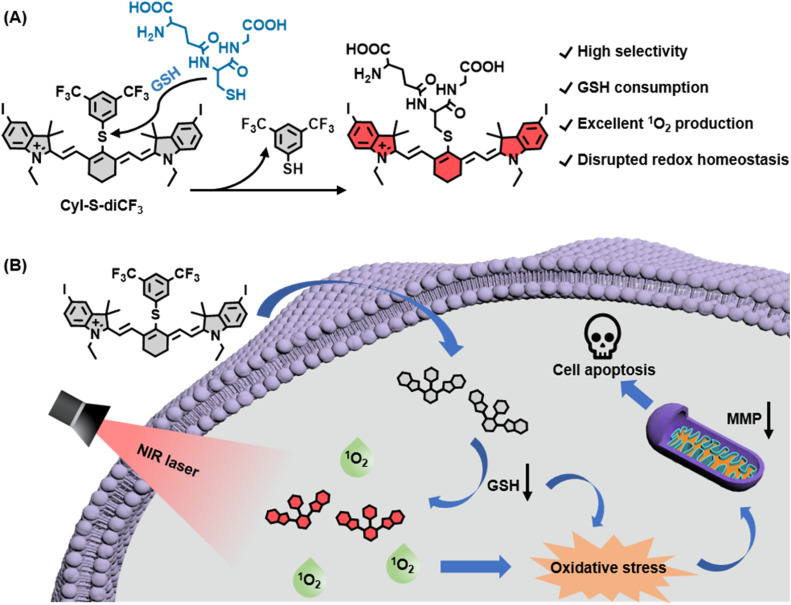
(A) Chemical structure and proposed activation mechanism of CyI-S-diCF_3_ mediated by GSH. (B) Illustrations of CyI-S-diCF_3_ for GSH depletion-assisted enhanced photodynamic therapy through breaking the redox homeostasis of tumor cells.

In this work, we developed a novel GSH-activated NIR photosensitizer (denoted as CyI-S-diCF_3_) for GSH depletion-assisted enhanced photodynamic therapy in tumor cells (Scheme 1). We first employed iodinated heptamethine cyanine dye as the NIR photosensitizer scaffold because of its superior ^1^O_2_ generation capability and biocompatibility. To confer the GSH responsiveness and depletion properties, we then incorporated an electron-withdrawing phenyl sulfide unit of 3,5-bis(trifluoromethyl)benzenethiol into the PS, which could more highly sensitive and selective to GSH than other related thiols. Within tumor cells, CyI-S-diCF_3_ was specifically activated by GSH, leading to the GSH decrease. The consumption of GSH not only elevated the intracellular ROS level, but also strengthened the ^1^O_2_ production efficiency under the NIR light irradiation, thus breaking the redox homeostasis in cancer cells. Furthermore, this disrupted redox homeostasis effectively induced mitochondrial membrane potential (MMP) dysfunction, followed by resulting in stronger and earlier apoptosis. Overall, we provide a new perspective on the design principle of organic photosensitizer to break the redox homeostasis of tumor cells.

## 2. Experimental section

### 2.1 Materials and instruments

All reagents and solvents used for the synthesis were acquired from commercial sources. 3,5-bis(trifluoromethyl)benzenethiol (BTBT, AR, 98%) was bought from Energy Chemical Co., Ltd. *N*, *N*-dimethylformamide (DMF, 99.8%) and dimethyl sulfoxide (DMSO, 99.9%) were purchased from Aladdin Chemistry Co., Ltd. *N*-Methylmaleimide (NMM) was obtained from Macklin Co., Ltd. Calcein-AM/PI detection kit, 2′,7′-dichloro-fluorescin diacetate (DCFH-DA) and JC-1 staining kit were supplied by Beyotime Institute of Biotechnology (Shanghai, China). Cell Counting Kit-8 (CCK-8) assay kit was acquired from GlpBio Technology Inc. Fetal bovine serum (FBS) and Roswell Park Memorial Institute 1640 medium (RPMI-1640) were purchased from GIBCO Co., Ltd.

The NMR spectra data were recorded on a Bruker Avance III 400 spectrometer, using TMS as the internal reference. High resolution mass spectra (HRMS) were performed using a LTQ Orbitrap XL spectrometer in the electrospray ionization (ESI) mode. Absorption and NIR fluorescence emission spectra were obtained on an UV-3600 spectrophotometer (Shimadzu) and a Hitachi F4700 fluorescence spectrometer, respectively. The cells imaging was conducted in an inverted fluorescence microscope (Carl Zeiss, Axio Observer A1). TLC analysis was performed on silica gel plates and column chromatography was conducted over silica gel (mesh 200–300) columns, obtained from the Jiangyou Yantai Co. Ltd (Shandong, China).

### 2.2 Synthesis of CyI-S-diCF_3_

To a dry 25 mL round bottom charged with CyI (30 mg, 0.033 mmol) dissolving in 3 mL anhydrous DMF, 3,5-bis(trifluoromethyl)benzenethiol (9.5 mg, 0.038 mmol) was added rapidly, and then the mixed solution was stirred for 12 h at room temperature under N_2_ atmosphere. After reaction finished, the mixture was evaporated in vacuum, and the crude product was purified by silica gel chromatography using CH_2_Cl_2_/CH_3_OH (15 : 1, v/v) as the eluent to obtain CyI-S-diCF_3_ as a dark green solid (20 mg, 54%). ^1^H NMR (400 MHz, DMSO-d_6_) *δ* 8.46 (d, *J* = 12 Hz, 2H), 7.98 (d, *J* = 4 Hz, 2H), 7.91 (s, 3H), 7.75 (dd, *J* = 8, 4 Hz, 2H), 7.26 (d, *J* = 8 Hz, 2H), 6.37 (d, *J* = 16 Hz, 2H), 4.20 (q, *J* = 8 Hz, 4H), 2.80 (t, *J* = 4 Hz, 4H), 2.00–1.85 (m, 2H), 1.41 (s, 12H), 1.25 (t, *J* = 8 Hz, 6H). ^13^C NMR (100 MHz, DMSO-d_6_) *δ* 171.50, 146.72, 144.36, 144.02, 141.92, 141.59, 137.69, 134.00, 132.01, 131.69, 126.77 (q, *J*_C–F_ = 9 Hz), 126.68, 124.65 (q, *J*_C–F_ = 212 Hz), 121.94, 119.99, 119.97 (q, *J*_C–F_ = 2 Hz), 114.03, 102.69, 90.17, 49.32, 27.44, 27.27, 26.39, 20.90, 12.59. ^19^F NMR (471 MHz, DMSO-d_6_) *δ* −61.67. HRMS (ESI) *m*/*z* calculated for C_42_H_41_F_6_I_2_N_2_S^+^ [M]^+^: 973.0979, found: 973.0967.

### 2.3 Fluorescence detection of GSH activity

CyI-S-diCF_3_ was dissolved in PBS/DMSO buffer (10 mM, pH 7.4, 7/3, v/v, 37 °C) with a concentration of 10 μM for the detection of GSH. With the addition of various concentrations of GSH (0–100 μM) to the CyI-S-diCF_3_ solution, the fluorescence intensity of the mixture at 823 nm was recorded after incubation at 37 °C for 5 min. (*λ*_ex_ = 780 nm). For time-dependent GSH response assay, upon GSH (100 μM) being added into the CyI-S-diCF_3_ solution at 37 °C, the FL spectra of the mixture at different time points (0–50 min) was measured in the range from 795 to 900 nm with the excitation of 780 nm. For the GSH inhibition experiment, CyI-S-diCF_3_ (10 μM) solution was treated with NMM (100 μM), followed by the addition of GSH (100 μM). After that, the mixture was further incubated for 5 min at 37 °C, and then the fluorescence intensity of above solution was measured immediately.

### 2.4 *In vitro* light-triggered singlet oxygen detection

ROS responsive probe 1,3-diphenylisobenzofuran (DPBF) was utilized to measure the singlet oxygen generation of CyI-S-diCF_3_. Especially, DPBF (3.0 μL, 20 mM) was added to the solution of CyI-S-diCF_3_ (10 μM) with or without GSH (100 μM) for 5 min in mixture of PBS/DMSO (10 mM, pH 7.4, 7/3, v/v, 1% tween 80, 37 °C). Then the mixed solution was irradiated with an 808 nm laser (0.33 W cm^−2^) for predetermined time intervals, and the absorption intensity of DPBF at 418 nm was recorded.

### 2.5 Determination of singlet oxygen quantum yield (*Φ*_Δ_)

The singlet oxygen quantum yield (*Φ*_Δ_) was tested by adjusted method according to literature. Typically, CyI-S-diCF_3_ was dissolved in PBS/DMSO buffer (10 mM, pH 7.4, 7/3, v/v, 1% tween 80, 37 °C) with 100 μM of GSH for 5 min, then the DPBF stock solution was added in above solution to adjust the absorbance at 418 nm was about 1.0. Afterward, UV-vis-NIR absorption spectra were recorded after the mixed solution was exposed to 808 nm laser irradiation with a power density of 0.33 W cm^−2^ for various time. Indocyanine green (ICG) was tested under the same experimental procedures as the reference. At last, the singlet oxygen quantum yield was calculated by the following equation:*Φ*_Δ_ = *Φ*_ICG_ × (*k*_(PSs)_ × *F*_(ICG)_)/(*k*_(ICG)_ × *F*_(PSs)_)where *k*_(PSs)_ and *k*_(ICG)_ were the decomposition rate constants of the absorbance at 418 nm of DPBF in the presence of the photosensitizer and ICG, respectively. *Φ*_Δ_ represents the singlet oxygen quantum yield of the tested photosensitizer; *Φ*_ICG_ represents the singlet oxygen quantum yield of ICG (*Φ*_ICG_ = 0.2% in water); *F* is the correction factor which is calculated by the following equation:*F* = 1 − 10^−O.D.^

O.D. is the absorbance of the mixture at 808 nm.

### 2.6 Extracellular depletion of GSH

Briefly, GSH (100 μM) was mixed with PBS or CyI-S-diCF_3_ (10 μM) in PBS/DMSO buffer (10 mM, pH 7.4, 7/3, v/v). After being incubated at 37 °C for 60 min, 200 μL of the above suspension and 1.0 μL of the DTNB solution (10 mM) were added into the working solution and further incubated for 5 min. Afterward, the absorbance at 412 nm was recorded *via* a UV-vis spectrophotometer.

### 2.7 Cell culture and *in vitro* cytotoxicity

Murine breast cancer cell (4T1) was obtained from the Institute of Basic Medical Sciences (IBMS) of the Chinese Academy of Medical Sciences. Cells were cultured in RPMI-1640 medium supplemented with 10% FBS with 5% CO_2_ at 37 °C. The cytotoxicity was measured using a standard CCK-8 assay. Typically, 4T1 cells were seed into 96-well microplates at an initial density of 3.5 × 10^3^ cells/well. After 24 h of adherence and stable growth, the cells were incubated with RPMI-1640 medium containing different concentrations of CyI-S-diCF_3_ for 4 h, then irradiated by 808 nm laser (1.0 W cm^−2^) for 5 min. As a control, a similar assay without light irradiation was performed. After further incubation for 2 h, CCK-8 assay was performed according to the protocol.

### 2.8 Dead/live cell co-staining

Firstly, 4T1 cells (3.5 × 10^3^) were seeded into the 96-well plate and cultured for 24 h. After reaching 80% confluence, the cells were treated with CyI-S-diCF_3_ (4.0 μM) at 37 °C for 4 h and then irradiated by 808 nm laser (1.0 W cm^−2^) for 5 min. Cells without irradiation were utilized as controls. Afterwards, the cells were co-stained with calcein AM and PI for 30 min and imaged by the fluorescence microscope, where live cells were stained in green and dead cells in red, respectively.

### 2.9 Intracellular ROS detection by DCFH-DA

Reactive Oxygen Species Assay Kit was used according to the manufacture instruction. Briefly, 4T1 cells were firstly seeded into 96-well plates with the cell density of 3 × 10^3^ cells per well and cultured for 24 h. Afterwards, the medium was replaced by fresh RPMI-1640 medium (control group, 808 nm laser group) or 4.0 μM of CyI-S-diCF_3_ (CyI-S-diCF_3_ group, CyI-S-diCF_3_ + 808 nm group) and incubated for another 4 h, followed by treating with 10 μM DCFH-DA for 30 min at 37 °C. After that, the cells were irradiated with 808 nm laser for 5 min at a power density of 1.0 W cm^−2^. Fluorescence images were captured by inverted fluorescence microscope.

### 2.10 Detection of mitochondrion membrane potential by JC-1 probe

3 × 10^3^ 4T1 cells were regularly seeded and cultured in 96-well plates for 24 h at 37 °C. Then, cells were incubated with CyI-S-diCF_3_ (4.0 μM) for 4 h and blank medium as negative control groups. After that, the cells in light groups were exposed to 808 nm laser (1.0 W cm^−2^) for 5 min, and then all the cells were cultured for another 2 h. Subsequently, the cells were stained by JC-1 kit according to the instruction manual. Ultimately, fluorescence microscope was applied to determine the mitochondrion membrane potential.

### 2.11 Evaluation of intracellular GSH depletion in 4T1 cells

According to the method in the previous literature with some modifications, 4T1 cells were seeded in 24-well plates at a density of 5 × 10^4^ cells per well and cultured for 24 h. The cells were treated with PBS or CyI-S-diCF_3_ (4.0 μM) for 4 h at 37 °C. Subsequently, all the cells were digestive by trypsin–EDTA and centrifuged at 1000 rpm for 4 min. Then 80 μL of Triton-X-100 lysis buffer (0.4%) was used to lyse the cells on ice for 20 min. Afterward, the lysates were centrifuged at 6000 rpm for 5 min, and 50 μL of supernatant was mixed with DTNB (150 μL, 0.5 mM). Finally, the absorbance (412 nm) of the solutions was measured using a microplate reader.

### 2.12 Statistical analysis

All data were expressed in this study as mean result ± standard deviation (S.D.). The statistical analysis was performed using Matlab software. The statistical differences between two groups were conducted by unpaired Student's two-sided *t* test analysis. Asterisks indicate significant differences (*P < 0.05, **P < 0.01, ***P < 0.001, ****P < 0.0001).

## 3. Result and discussion

### 3.1 Synthesis and activatable property of CyI-S-diCF_3_

**Fig. 1:**
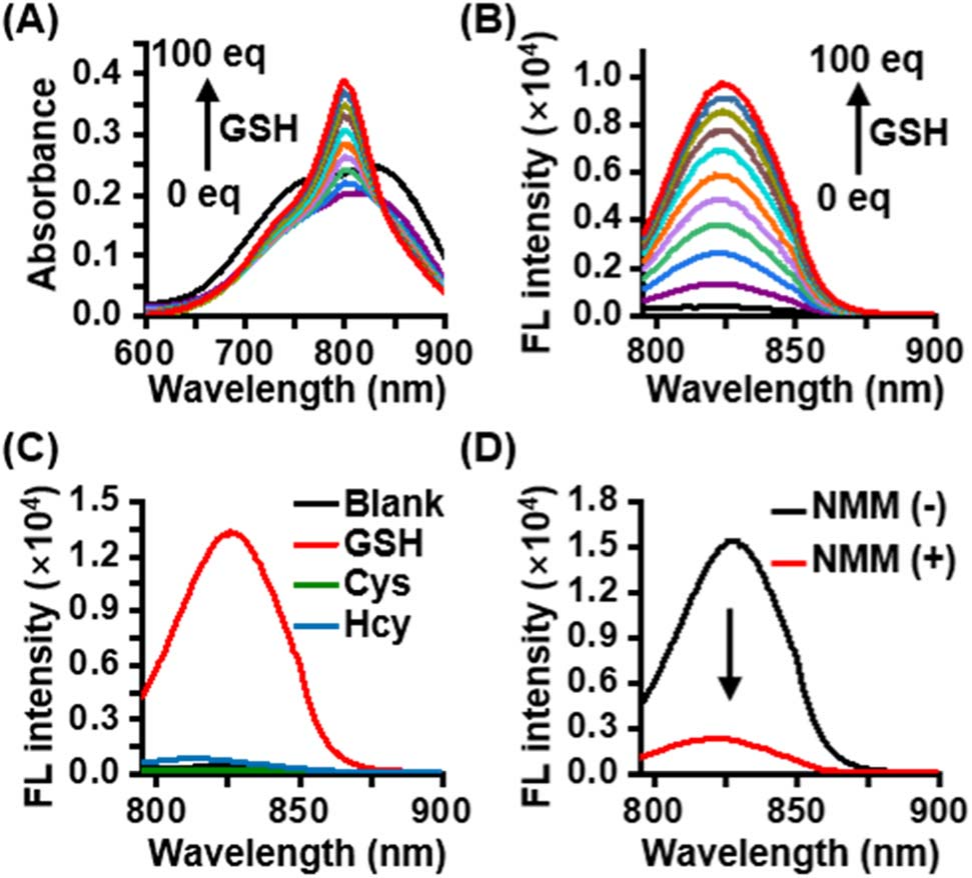
(A) The UV-vis absorption spectra and (B) fluorescence spectra of CyI-S-diCF_3_ (10 μM) after incubation with different concentrations of GSH (0–100 μM) in PBS (10 mM, pH 7.4, 30% DMSO). (C) Fluorescence responses of CyI-S-diCF_3_ (10 μM) with the addition of various amino acids (100 μM) in PBS (10 mM, pH 7.4, 30% DMSO) at 37 °C for 5 min (blank, GSH, Cys, Hcy). (D) The NIR-I fluorescence spectra of CyI-S-diCF_3_ (10 μM) in the presence of GSH (100 μM) with or without addition of NMM (100 μM) in PBS (10 mM, pH 7.4, 30% DMSO) at 37 °C for 5 min.

CyI-S-diCF_3_ was easily prepared through the sulfhydryl nucleophilic substitution reaction of chlorine atoms on iodinated heptamethine cyanine dye (CyI) by 3,5-bis(trifluoromethyl)benzenethiol (BTBT) (Fig. S1†). The intermediate of CyI was synthesized according to the previous ref. 41 The final product was fully characterized by ^1^H NMR, ^13^C NMR, ^19^F NMR and high-resolution mass spectrum (ESI-HRMS). Typically, CyI-S-diCF_3_ generated 13 proton signals in the downfield region as shown in the Fig. S6† from aromatic carbon and polymethine bridge, while the remaining 28 proton signals were found in the aliphatic region, indicating the accuracy of the structure. In addition, the coupling constant of polymethine bridge was calculated to be 16 Hz, which was larger than 15 Hz, suggested that CyI-S-diCF_3_ was mainly existing in the E-geometry. Moreover, ^13^C NMR and ^19^F NMR spectra have furtherly confirmed that the structure possessed the CF_3_ functional group (Fig. S7 and S8†), and ESI-HRMS also showed an expected molecular weight (Fig. S9†). Thus, those characterization results were consistent with the proposed structure. To investigate the sensitivity toward GSH, photochemical properties of CyI-S-diCF_3_ was determined based on GSH concentration and response time in PBS/DMSO mixtures (10 mM, pH 7.4, 7/3, v/v). Upon the addition of GSH (0–100 eq.) to the CyI-S-diCF_3_ solution (10 μM), the absorbance at 798 nm increased gradually due to the formation of a water-soluble product triggered by GSH (Fig. 1A). Meanwhile, the fluorescence intensity of CyI-S-diCF_3_ at 823 nm increased proportionally to the GSH concentration (Fig. 1B). This remarkable enhancement emission could be attributed to the restriction of the photoinduced electron-transfer (PET) effect from BTBT moiety to heptamethine. Of note, a good linear relationship was observed between the fluorescence intensities of CyI-S-diCF_3_ and the GSH concentration (Fig. S2†). In addition, the time-dependent fluorescence changes of CyI-S-diCF_3_ with various concentration of GSH was also investigated, where the intensity variations rapidly increased and reached a plateau within 50 min (Fig. S3 and S4†). Although CyI-S-diCF_3_ exhibited a similar sensitivity toward GSH compared with the reported NIR photosensitizer, it possessed a longer excitation wavelength (700–900 nm) than other one, suggesting its potential to precise photodynamic therapy.^42,43^

Subsequently, the selectivity of CyI-S-diCF_3_ was evaluated against similar biothiols such as l-cysteine (Cys) and l-homocysteine (Hcy). As shown in Fig. 1C, only GSH treated group showed a significant change in fluorescence intensity, while Cys and Hcy treated group could not exhibited obvious fluorescence signal, thus suggesting a higher GSH-specific responsiveness of CyI-S-diCF_3_ then some reported AIE based GSH activatable photosensitizers (*e.g.*, TPEPY-S-Fc), as it could not able to distinct the GSH from other closely related thiols (*e.g.*, Cys, Hcy).^44^ It is worth noting that the activation efficiency of CyI-S-diCF_3_ by GSH was significantly decreased after the addition of *N*-methylmaleimide (NMM, a GSH inhibitor) (Fig. 1D), further implying that CyI-S-diCF_3_ was a GSH-specific activation probe.

### 3.2 Extracellular singlet oxygen (^1^O_2_) generation and GSH depletion

**Fig. 2:**
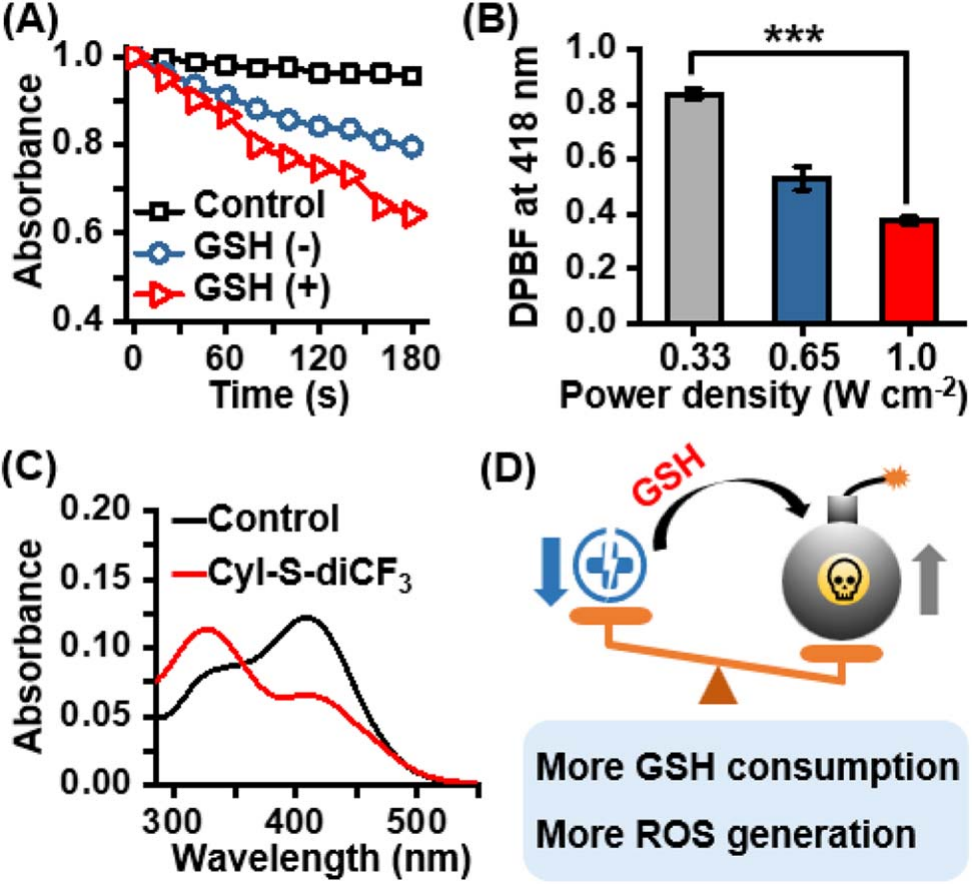
(A) ROS generation from CyI-S-diCF_3_ (10 μM) with or without GSH (100 μM) using DPBF as a probe under 808 nm irradiation (0.33 W cm^−2^, 3 min). (B) Compare the attenuation of the maximum absorbance of DPBF at 60 s of irradiation with 808 nm laser through various power density. Data are presented as the mean ± SD (*n* = 3). *P < 0.05, **P < 0.01, ***P < 0.001, and ****P < 0.0001. (C) GSH-depletion abilities in the absence and presence of CyI-S-diCF_3_ (4.0 μM) with indicator DTNB, respectively. (D) Schematic illustration of oxidative stress strengthened by GSH depletion and enhanced singlet oxygen production efficiency.

After confirming the excellent selectivity of CyI-S-diCF_3_ to GSH, the ^1^O_2_ generation capability of CyI-S-diCF_3_ in the absence and presence of GSH under 808 nm irradiation was then assessed by using 1,3-diphenylisobenzofuran (DPBF) as the ^1^O_2_ trapping agent. Compare to the DPBF group, the PBS/DMSO solution with DPBF and CyI-S-diCF_3_ still showed a slightly reduction in DPBF absorbance at 418 nm under the 808 nm irradiation. Nevertheless, the absorption intensity of DPBF decreased rapidly with a continuous irradiation time from 0 to 180 s after CyI-S-diCF_3_ was pretreated with GSH at 37 °C for 5 min, indicating a superior ^1^O_2_ production efficiency (Fig. 2A). Therefore, it is reasonable to infer that GSH-activated CyI-S-diCF_3_ could simultaneously favor radiative decay pathway and ISC transitions rate. The ^1^O_2_ generation efficiency was further quantified by calculating the singlet oxygen quantum yield (*Φ*_Δ_) of CyI-S-diCF_3_ before and after GSH response through using ICG as the reference (*Φ*_Δ_ = 0.2% in water). Significantly, CyI-S-diCF_3_ exhibited a higher quantum yield of 0.66% after triggered by reaction with GSH, while the CyI-S-diCF_3_ in absence of GSH resulted in the relative lower quantum yields of 0.45% (Fig. S5†), indicating that CyI-S-diCF_3_ could consume the GSH and followed by enhancing the ^1^O_2_ generation capability. Moreover, the ^1^O_2_ generation efficiency of CyI-S-diCF_3_ treating with GSH displayed a significant positive correlation with laser density (Fig. 2B), suggesting that laser power density could serve as an “accelerator” to promote the ^1^O_2_ production.

In light of the good GSH-specific responsiveness, the capacity of CyI-S-diCF_3_ to deplete GSH was evaluated by using the 5,5′-dithiobis-(2-nitrobenzoic acid) (DTNB), which could react with GSH to produce yellow derivative 5-thio-2-nitrobenzoic acid (TNB). As illustrated in Fig. 2C, the absorbance peak of TNB at 412 nm decreased distinctly with the addition of CyI-S-diCF_3_, indicating that the GSH could be effectively consumed by CyI-S-diCF_3_. Taken all together, the GSH-activated CyI-S-diCF_3_ with GSH depletion and enhanced ^1^O_2_ generation properties could be used as a ROS amplifier to disrupt the redox homeostasis in microenvironment of cancer cells (Fig. 2D).

### 3.3 *In vitro* cytotoxicity evaluation

**Fig. 3:**
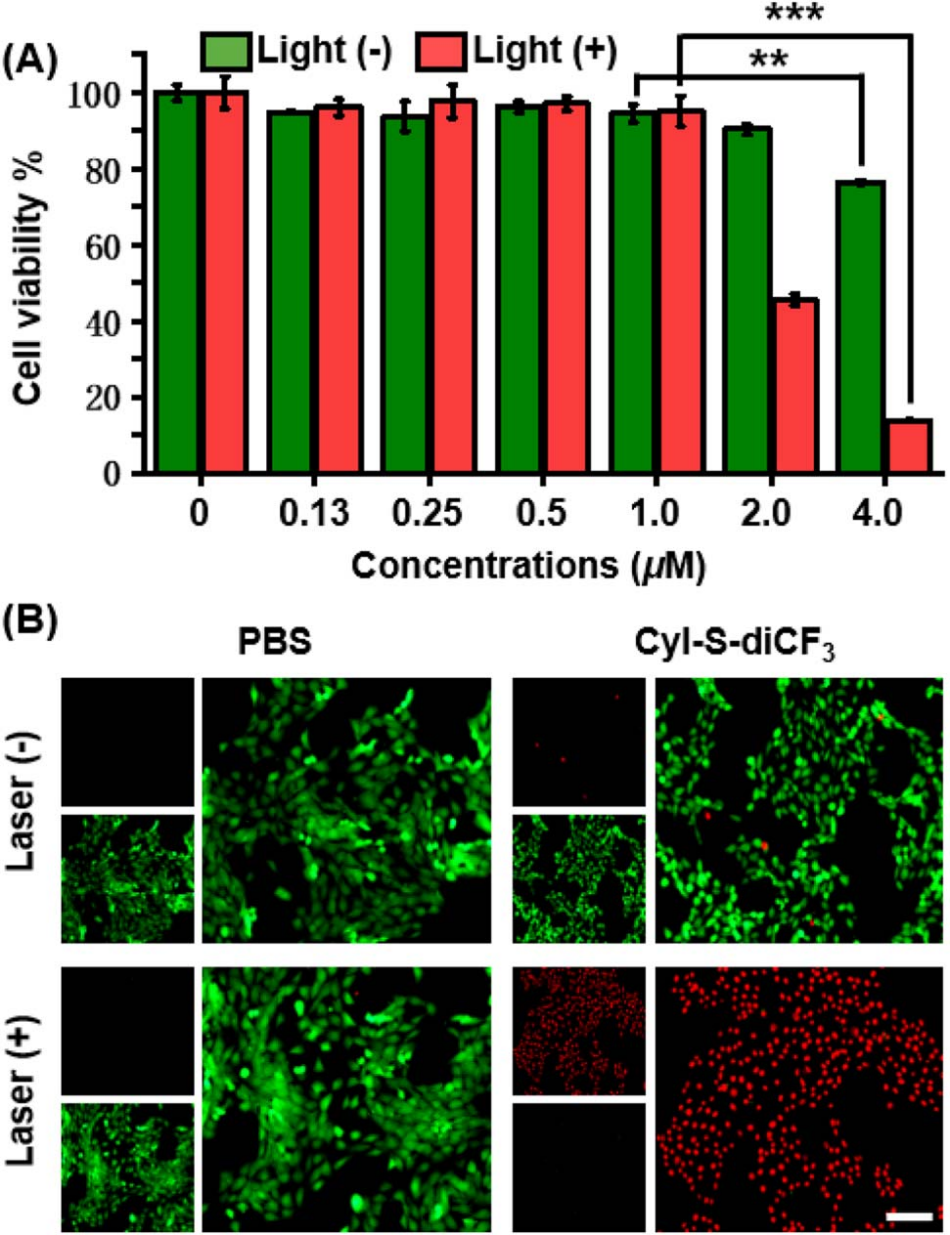
*In vitro* antitumor effects of CyI-S-diCF_3_. (A) Cell viabilities of 4T1 cells treated with CyI-S-diCF_3_ at various concentrations in the dark or under 808 nm laser irradiation (1.0 W cm^2^, 5 min). Data are presented as the mean ± SD (*n* = 3). *P < 0.05, **P < 0.01, ***P < 0.001, and ****P < 0.0001. (B) Calcein AM (green) and propidium iodide (red) co-staining fluorescence imaging of 4T1 cells after different treatments. 808 nm light irradiation (1.0 W cm^2^, 5 min) was conducted after cells were incubated with CyI-S-diCF_3_ (4.0 μM) for 4 h. Scale bar: 100 μm.

The anticancer effect of CyI-S-diCF_3_ against 4T1 cells was investigated using a standard CCK-8 assay. Tumor cells were treated with various concentrations of CyI-S-diCF_3_ in dark or under 808 nm light irradiation, and then the cell viability was measured. As shown in Fig. 3A, CyI-S-diCF_3_ exhibited negligible toxicity to cells at the low concentrations in dark, whereas obvious cytotoxicity (below 80%) was noted at concentrations up to 4.0 μM, which was partially attributed to elevation of ROS levels in tumor cells cause by GSH consumption. In addition, the cytotoxicity of CyI-S-diCF_3_ from the light group showed a significant concentration dependence. The viability of 4T1 cell dropped to almost 10% when the cells pre-treated with CyI-S-diCF_3_ (4.0 μM) were exposed to 808 nm irradiation (1.0 W cm^−2^, 5 min), suggesting its great ROS generation efficiency.

Subsequently, live/dead cell staining assay was conducted to visualize the PDT efficacy of CyI-S-diCF_3_ (Fig. 3B). Calcein acetoxymethyl ester (calcein-AM) was hydrolyzed into calcein and emitted green fluorescence in living cells, whereas propidium iodide (PI) only exhibited red fluorescence in dead cells duo to its poor cell membrane permeability. The cells treated with PBS and PBS with light showed a bright green fluorescence, indicating light alone did not harm cells. In contrast, some red fluorescence was observed in 4T1 cells incubated with CyI-S-diCF_3_ in the dark, demonstrating slightly killing effect causing by the reduction of GSH content in cells. Moreover, cells cultured with CyI-S-diCF_3_ under 808 nm irradiation (1.0 W cm^−2^, 5 min) displayed intense red fluorescence and negligible green fluorescence, again illustrating the strong antitumor efficacy of CyI-S-diCF_3_. These results showed that CyI-S-diCF_3_ can be used as a highly effective photosensitizer to induce tumor cell death triggered by intracellular GSH depletion and sufficient ^1^O_2_ production under light.

### 3.4 Intracellular ROS and GSH detection

**Fig. 4:**
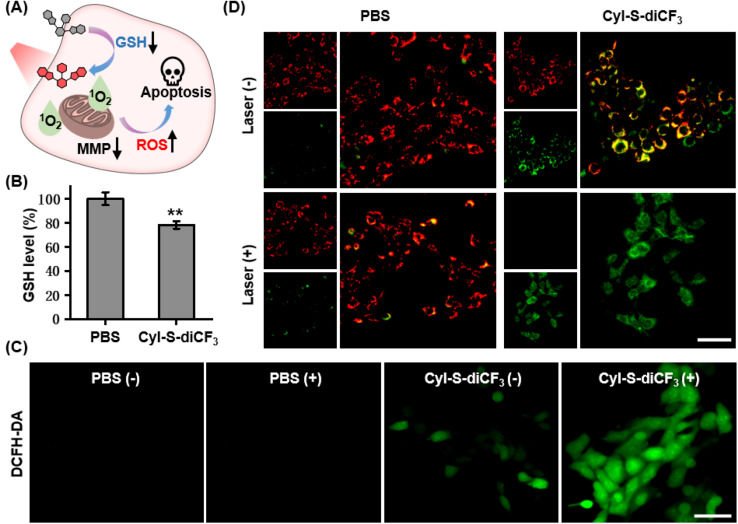
(A) Schematic illustration of cell apoptosis induced by GSH-depleted photosensitizers and light-triggered singlet oxygen production to amplify the oxidative stress. (B) Cellular GSH levels of 4T1 cells after treatment of CyI-S-diCF_3_ for 4 h. Data are presented as the mean ± SD (*n* = 3). *P < 0.05, **P < 0.01, ***P < 0.001, and ****P < 0.0001. (C) Detection of intracellular ROS generation by DCFH-DA in 4T1 cells after different treatments: (1) PBS only, (2) PBS with 808 nm laser, (3) CyI-S-diCF_3_ only, (4) CyI-S-diCF_3_ with 808 nm laser. (D) Mitochondrial membrane potential monitored by using JC-1 dye after incubation without and with CyI-S-diCF_3_ (4.0 μM) for 4 h, followed by 808 nm laser irradiation (1.0 W cm^2^, 5 min). Scale bar: 50 μm.

Based on the good GSH specificity, CyI-S-diCF_3_ could exhibited intense fluorescence signal and elevate ^1^O_2_ generation efficiency after activated by GSH, following disrupting the redox homeostasis in tumor cells. The resulting oxidative stress further promoted mitochondrial membrane potential damage, leading to cell apoptosis (Fig. 4A). Encouraged by the GSH depletion property of CyI-S-diCF_3_ at extracellular level, the intracellular GSH content was firstly examined (Fig. 4B). The untreated control group showed a negligible change in GSH level, whereas the cells incubated with CyI-S-diCF_3_ could induce approximately 22% reduction of GSH, suggesting that CyI-S-diCF_3_ could promote the consumption of GSH in tumor cells.

Next, in order to assess the GSH accelerated photodynamic effect, the ROS generation efficiency of CyI-S-diCF_3_ in 4T1 cells was evaluated using DCFH-DA as the detection probe. As shown in Fig. 4C, weak green fluorescence was clearly observed in 4T1 cells with the treatment of CyI-S-diCF_3_ in dark, indicating the down-regulation of GSH and the enhancement of ROS. Thus, compared to other GSH activatable NIR photosensitizers, CyI-S-diCF_3_ exhibited the significant GSH consumption property, implying that it could be used as an inducer to break the redox homeostasis in tumor cells.^43,45^ Moreover, the cells treated with CyI-S-diCF_3_ under 808 nm irradiation (1.0 W cm^2^, 5 min) exhibited intense green fluorescence, implying the presence of large amounts of ^1^O_2_. However, no green fluorescence signal was detected in the PBS treated cells in dark or under light. These results firmly demonstrated that GSH and light irradiation could sequentially manipulate the ROS production efficiency of CyI-S-diCF_3_ in tumor cells. The mitochondrial membrane potential (MMP) assay was then performed as it could be a significant index to evaluate the extent of the mitochondrial destruction. From the Fig. 4D, we clearly found that the cells in control group displayed solely strong red fluorescence because of its good integrity in mitochondria. However, both intensive green fluorescence (JC-1 monomers) and red fluorescence (JC-1 aggregates) were observed in cells treated with CyI-S-diCF_3_ in dark, suggesting its partial potential loss to the mitochondrial membrane. Notably, under 808 nm light irradiation (1.0 W cm^2^, 5 min), the cells incubated with CyI-S-diCF_3_ exhibited strong green fluorescence but weak red fluorescence, indicating a severely decrease in mitochondrial membrane potential. In short, CyI-S-diCF_3_ could be served as a smart photosensitizer to effectively induce cells apoptosis causing by GSH down-regulation and enhancement of ^1^O_2_ generation.

## 4. Conclusion

In summary, we reported a new GSH activated photosensitizer based on a heptamethine cyanine scaffold. The single-atom replacement of hydrogen for iodine significantly enhanced the photodynamic efficiency of heptamethine cyanine due to the heavy-atom effect (HAE). More importantly, the recognition units of 3,5-bis(trifluoromethyl)benzenethiol on photosensitizer showed a higher sensitivity and selectivity toward GSH then other closely related biothiols. After triggering by cellular GSH, CyI-S-diCF_3_ could switch to “on” state and produced abundant of toxic ^1^O_2_ under 808 nm light irradiation. This high-level of ROS generation and antioxidant GSH elimination could effectively break the cellular redox homeostasis, leading to augmented oxidative stress. *In vitro* experiment demonstrated excellent GSH depletion-assisted enhanced PDT effects of the CyI-S-diCF_3_ against tumor cells. Thus, this study may provide a new strategy for designing activatable NIR photosensitizer with disrupted redox homeostasis property.

## Conflicts of interest

There are no conflicts to declare.

## Supplementary Material

RA-013-D3RA04074G-s001
