# Supplementary material for: Engineering of a GSH activatable photosensitizer for enhanced photodynamic therapy through disrupting redox homeostasis
Source: RSC Adv. 2023 Jul 25;13(32):22367–74. doi: 10.1039/d3ra04074g (PMC10366568; doi:10.1039/d3ra04074g)
Supplement: RA-013-D3RA04074G-s001 [file RA-013-D3RA04074G-s001.pdf]

Electronic Supplementary Material (ESI) for RSC Advances.
This journal is © The Royal Society of Chemistry 2023

Engineering of a GSH activatable photosensitizer for enhanced photodynamic therapy through disrupting redox homeostasis

Datian Fu [a, 1], Yan Wang [a, 1], Kaiwen Lin [a], Liangjiu Huang [b], Jin Xu [c], Haimei Wu [b, *]

[a] Department of Pharmacy, Hainan women and children's medical center, Haikou, Hainan 570100, China.

[b] Department of Clinical Pharmacy, Hainan Cancer Hospital, Haikou, Hainan 570100, China.

[c] Pharmaceutical and bioengineering school, Hunan Chemical Vocational Technology College, Zhuzhou,412006, China

* Corresponding authors:

Haimei Wu, E-mail: wu634202863@126.com

[1] These authors contribute equally to this work.

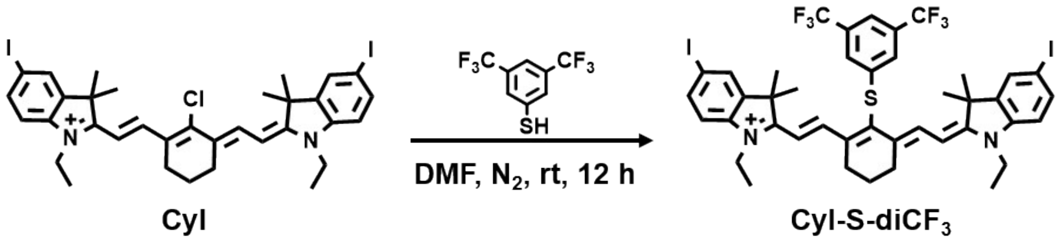

Fig. S1 The synthesis route of CyI-S-diCF_3.

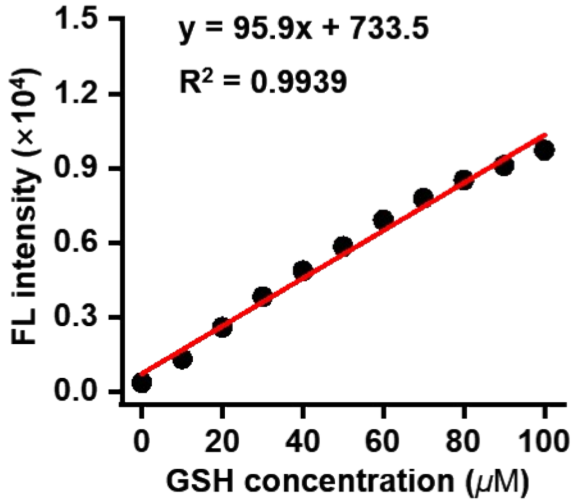

Fig. S2 The linear plot of fluorescence intensity of CyI-S-diCF_3 (10 μM) in the presence of various concentrations of GSH (0-100 μM).

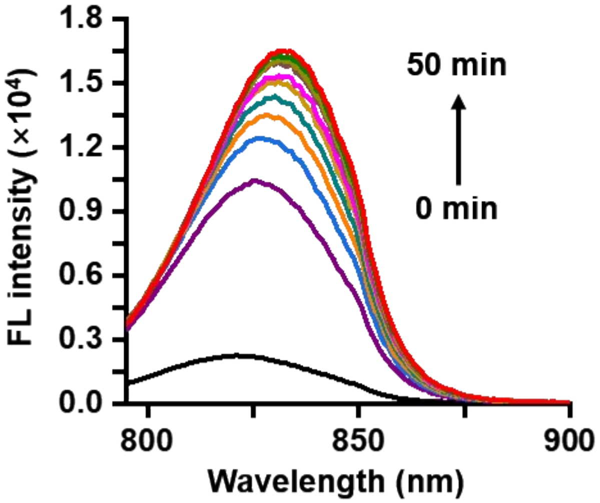

Fig. S3 Time-dependent NIR fluorescence spectra of CyI-S-diCF$_3$ (10 μM) in PBS (10 mM, pH 7.4, 30% DMSO) after incubation with GSH (100 μM) at 37 °C for varied time.

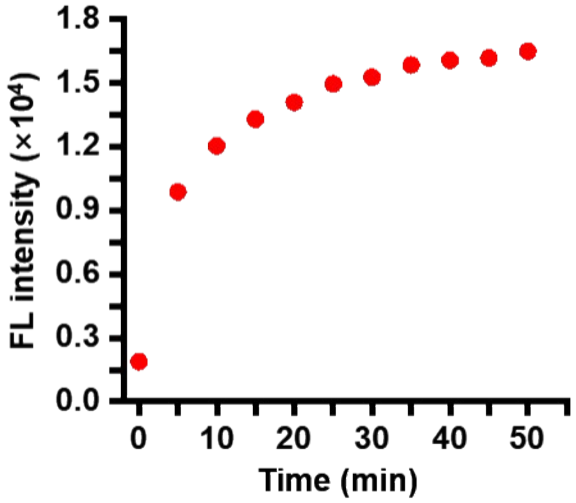

Fig. S4 The corresponding plot of fluorescence intensity of CyI-S-diCF$_3$ after treated with GSH (100 μM) at 37 °C within 50 min.

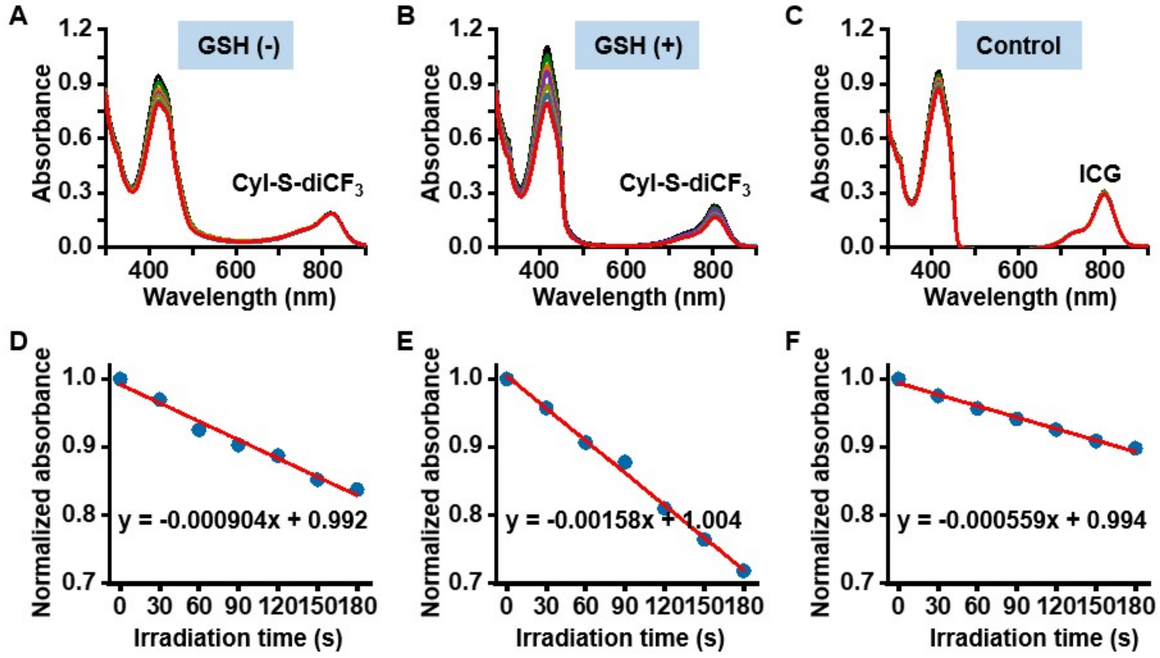

Fig. S5 The singlet oxygen quantum yield calculation. DPBF absorbance change at 418 nm induced by (A) CyI-S-diCF$_3$ without GSH, (B) CyI-S-diCF$_3$ treated with GSH (100 μM), and (C) ICG after 808 nm

irradiation. (D-F) The corresponding linear calibration curve for the absorbance of DPBF to illumination time.

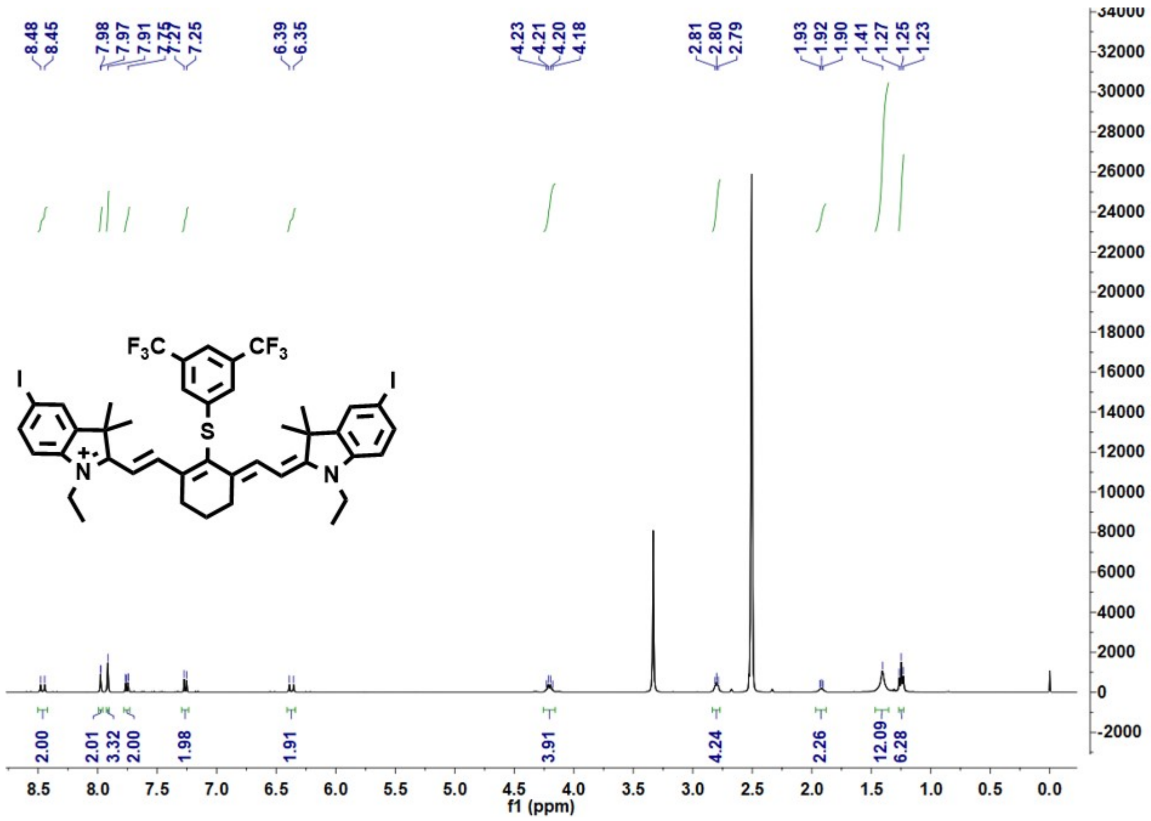

Fig. S6 ^{1}H NMR spectra of CyI-S-diCF$_3$.

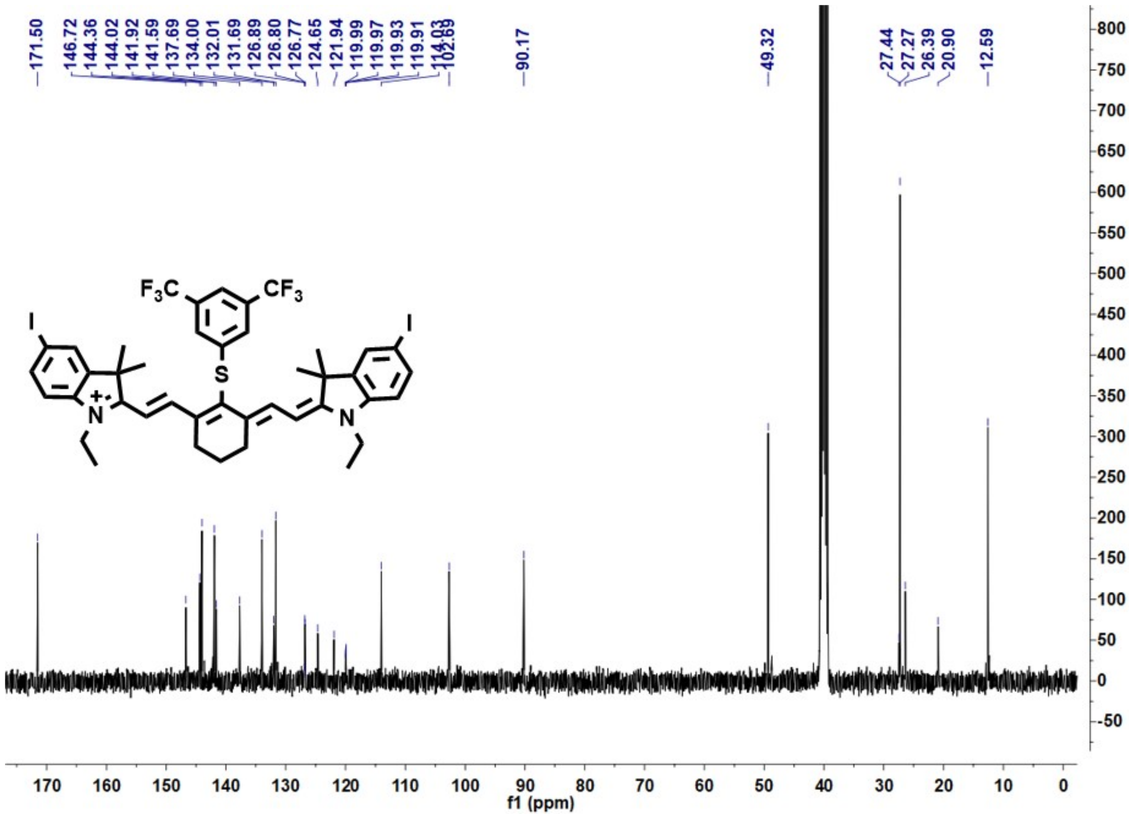

Fig. S7 ^{13}C NMR spectra of CyI-S-diCF_3.

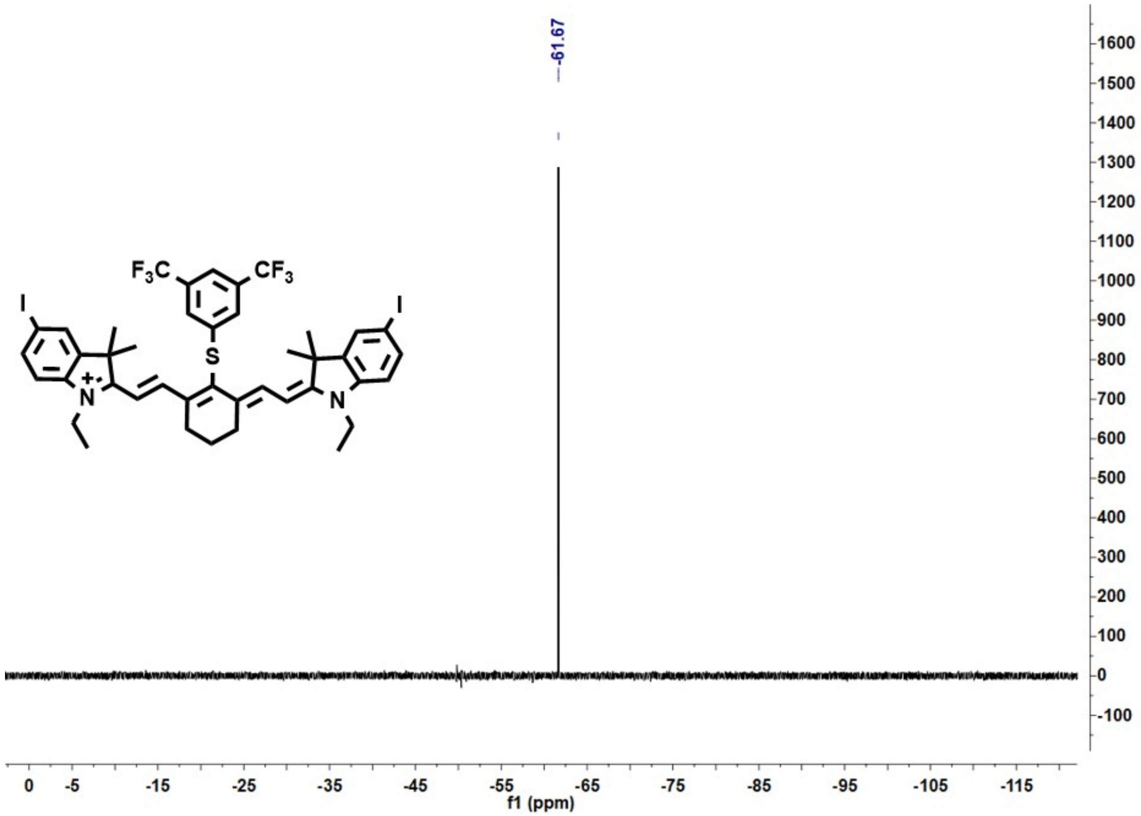

Fig. S8 ^{19}F NMR spectra of CyI-S-diCF_3.

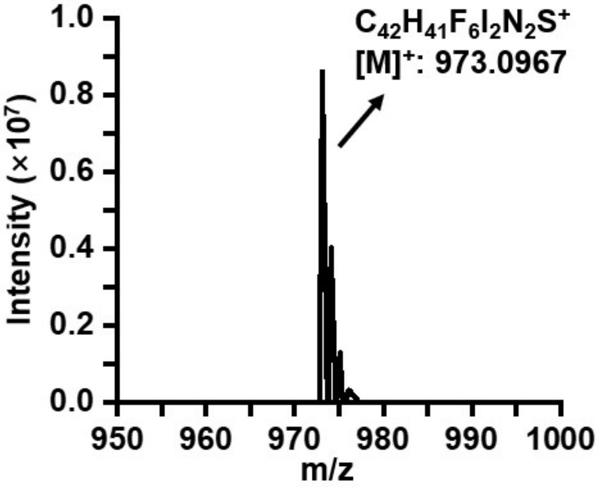

Fig. S9 ESI-HRMS spectrum of CyI-S-diCF_3.
